# Insulin-Like Growth Factor-1 and Neuroinflammation

**DOI:** 10.3389/fnagi.2017.00365

**Published:** 2017-11-03

**Authors:** Jose L. Labandeira-Garcia, Maria A. Costa-Besada, Carmen M. Labandeira, Begoña Villar-Cheda, Ana I. Rodríguez-Perez

**Affiliations:** ^1^Laboratory of Neuroanatomy and Experimental Neurology, Department of Morphological Sciences, CIMUS, University of Santiago de Compostela, Santiago de Compostela, Spain; ^2^Networking Research Center on Neurodegenerative Diseases (CIBERNED), Madrid, Spain; ^3^Department of Clinical Neurology, Hospital Alvaro Cunqueiro, University Hospital Complex, Vigo, Spain

**Keywords:** aging, angiotensin, astrocytes, estrogen, IGF-1, insulin, microglia, neurodegeneration

## Abstract

Insulin-like growth factor-1 (IGF-1) effects on aging and neurodegeneration is still controversial. However, it is widely admitted that IGF-1 is involved in the neuroinflammatory response. In peripheral tissues, several studies showed that IGF-1 inhibited the expression of inflammatory markers, although other studies concluded that IGF-1 has proinflammatory functions. Furthermore, proinflammatory cytokines such as TNF-α impaired IGF-1 signaling. In the brain, there are controversial results on effects of IGF-1 in neuroinflammation. In addition to direct protective effects on neurons, several studies revealed anti-inflammatory effects of IGF-1 acting on astrocytes and microglia, and that IGF-1 may also inhibit blood brain barrier permeability. Altogether suggests that the aging-related decrease in IGF-1 levels may contribute to the aging-related pro-inflammatory state. IGF-1 inhibits the astrocytic response to inflammatory stimuli, and modulates microglial phenotype (IGF-1 promotes the microglial M2 and inhibits of M1 phenotype). Furthermore, IGF-1 is mitogenic for microglia. IGF-1 and estrogen interact to modulate the neuroinflammatory response and microglial and astrocytic phenotypes. Brain renin-angiotensin and IGF-1 systems also interact to modulate neuroinflammation. Induction of microglial IGF-1 by angiotensin, and possibly by other pro-inflammatory inducers, plays a major role in the repression of the M1 microglial neurotoxic phenotype and the enhancement of the transition to an M2 microglial repair/regenerative phenotype. This mechanism is impaired in aged brains. Aging-related decrease in IGF-1 may contribute to the loss of capacity of microglia to undergo M2 activation. Fine tuning of IGF-1 levels may be critical for regulating the neuroinflammatory response, and IGF-1 may be involved in inflammation in a context-dependent mode.

## Introduction

Insulin-like growth factor-1 (IGF-1) is a protein produced in several organs, such as gonads, muscle, bones, liver, gut and brain and is also present in plasma. IGF-1 signals primarily via IGF-1 receptors (IGF-1R), but IGF-1 can act also through the insulin receptor. IGF-1 is actively transported to the central nervous system (CNS) from plasma through the choroid plexus (Carro et al., 2000; Santi et al., 2017), and it is also locally produced in the brain by neurons and glial cells (Quesada et al., 2007; Suh et al., 2013; Rodriguez-Perez et al., 2016). IGF-1 has multiple effects in the CNS, regulating early brain development, myelination, synapse formation, adult neurogenesis, production of neurotransmitters and cognition (Nieto-Estévez et al., 2016; Wrigley et al., 2017). Furthermore, it is usually considered that IGF-1 is a potent neuroprotective compound (Carro et al., 2003; Tien et al., 2017), and that this is, at least partially, due to inhibition of neuroinflammation (Sukhanov et al., 2007; Park et al., 2011). Consistent with this, a decrease in IGF-1 signaling has been related with neurodegeneration, depressive disorders and other brain diseases, in which IGF-1 has been suggested as a possible therapy (Torres Aleman, 2012; Guan et al., 2013). Several decades ago, circulating GH and IGF-1 have been shown to decrease with aging. However, the possible relationship between IGF-1 effects and aging is still controversial, and opposite concepts can be found in the literature. Both the increase in IGF-1 levels and the inhibition of the IG-1R signal appear to induce beneficial effects in the CNS, and exert either aging or anti-aging effects (Cohen and Dillin, 2008; Fernandez and Torres-Alemán, 2012; Sonntag et al., 2012).

This apparent contradiction has been related to different explanations. Mild decrease in IGF-1 signal may lead to mild metabolic changes that induce protective defenses against more intense and deleterious conditions associated to aging, finally leading to an increase in lifespan (Fernandez and Torres-Alemán, 2012; Sonntag et al., 2012). IGF-1 decrease may lead to increased resistance to oxidative stress (OS), mild mitochondrial dysfunction leading to hormesis (Troulinaki and Bano, 2012), or increased resistance to proteotoxicity (Cohen et al., 2009; George et al., 2017). It has also been proposed that deletion of IGF-1R may counteract possible deleterious IGF-1R signaling independently of IGF-1 (Torres Aleman, 2012). Inhibition of IGF-1-related tumor development may also result in increased longevity (Novosyadlyy and Leroith, 2012). We suggest that development of compensatory mechanisms against mild dysregulation of the neuroinflammatory response may also be involved. Actions of IGF-1 may be context-dependent (Fernandez and Torres-Alemán, 2012), and IGF-1 may be involved in inflammation in a context-dependent mode, which may explain controversial results on the role of IGF-1 in neuroinflammation.

In summary, it is known that IGF-1 plays a major role in regulation of brain cells in health and disease conditions, although this role is controversial and has not been totally clarified. However, it is widely admitted that IGF-1 levels increase in response to brain injury, and IGF-1 is involved in the neuroinflammatory response to injury. Since neuroinflammation plays a major role in brain aging and neurodegeneration, clarification of the role of IGF-1 in the neuroinflammatory process may shed light on the above mentioned controversy.

## IGF-1 and Neuroinflammation

In peripheral tissues, several studies have shown that IGF-1 regulates macrophagic functions, inhibits expression of pro-inflammatory cytokines and decreases disease progression (Sukhanov et al., 2007; Hijikawa et al., 2008). However, other studies concluded that IGF-1 has proinflammatory functions, as it was observed that IGF-1 increased chemotactic migration and TNF-α expression in macrophages (Renier et al., 1996). Conversely, proinflammatory cytokines such as TNF-α impaired IGF-1 signaling (Hotamisligil et al., 1993). Controversial results on effects of IGF-1 in neuroinflammation have also been reported. IGF-1 (Park et al., 2011) and IGF-1 gene transfer (Hung et al., 2007; Dodge et al., 2008) inhibited neuroinflammatory responses. However, other studies observed that inhibition of IGF-1R signaling decreased neuroinflammation and neuronal death in Alzheimer’s disease (AD) mice models: IGF1R-deficient mice were more resistant to amyloid-β oligomer-induced proteotoxicity, showing notably less activated astrocytes and less microgliosis (Cohen et al., 2009; George et al., 2017). As previously observed in peripheral tissues, TNF-α inhibited IGF-1 signaling in neurons (Venters et al., 1999).

The mechanisms responsible for the above mentioned effects are unclear. The anti-inflammatory properties of IGF-1 may be related to regulation of infiltration of inflammatory cells into tissues (Motani et al., 1996), including CNS, rather than a direct effect on the inflammatory cells (i.e., IGF-1 may act on the blood brain barrier; BBB). Consistent with this, IGF-1 ameliorated the breakdown of the BBB in a traumatic lesion of the spinal cord (Sharma, 2005), and in a model of multiple sclerosis (Liu et al., 1995). However, other studies showed that, IGF-1 enhanced BBB permeability and leukocyte infiltration (Pang et al., 2010): co-administration of IGF-1 with LPS further enhanced BBB permeability induced by LPS alone, while no change of BBB integrity was found in rats treated with IGF-1 alone. It was suggested that the effects of IGF-1 may depend on pathological conditions, and that in an acute inflammation, IGF-1 may have detrimental effects. However, several *in vitro* experiments revealed direct anti-inflammatory effects of IGF-1 on astrocytes and microglia (Bluthé et al., 2006; Palin et al., 2007). A simultaneous effect on the BBB and glial cells was also suggested (Bake et al., 2014).

**Figure 1:**
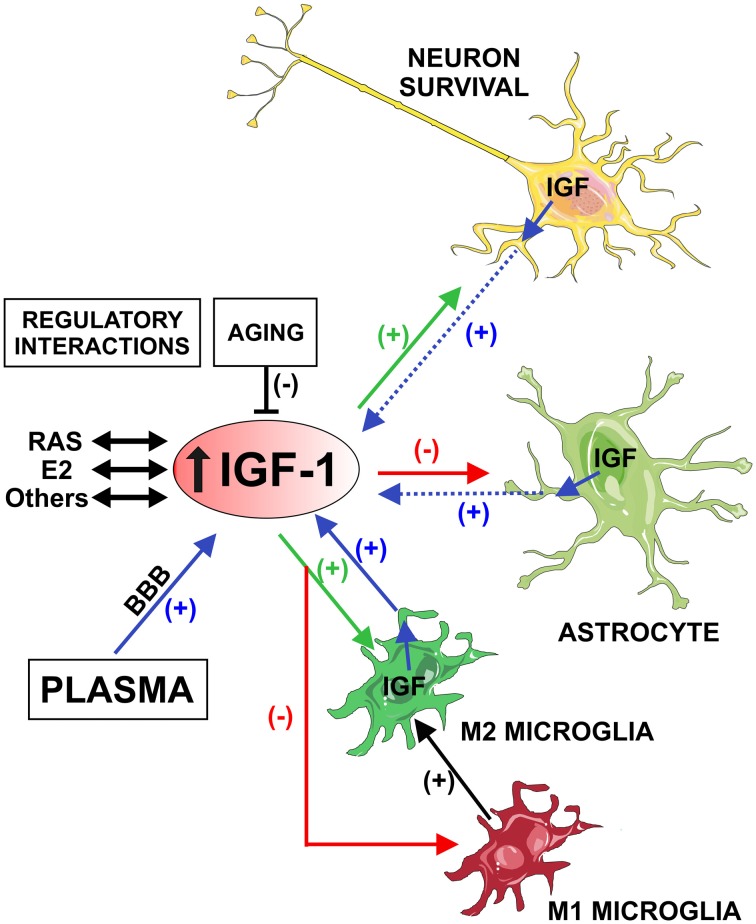
Schematic model showing the possible role of Insulin-like growth factor-1 (IGF-1) in neuroinflammation. IGF-1 is actively transported from plasma and locally produced in the brain by neurons and glial cells (blue arrows). Microglial cells are a major source of IGF-1(blue arrow) in comparison with astrocytes and neurons (dashed blue arrows). IGF-1 receptors are predominantly expressed in neurons and astrocytes, which appear to be targeted by IGF-1 in lesioned regions. IGF-1 promotes neuronal survival and the M2 microglial repair/regenerative phenotype (green arrows), and inhibits the astrocytic response to inflammatory stimuli and the M1 microglial phenotype (red arrows). Therefore, IGF-1 induces repression of the M1 microglial neurotoxic phenotype and enhancement of the transition to M2 (black arrow). Aging-related decrease in IGF-1 may contribute to the loss of capacity of microglia to undergo M2 activation, leading to an aging-related pro-inflammatory state. Brain IGF-1, estrogen and angiotensin interact to modulate the neuroinflammatory response. However, these regulatory mechanisms are impaired in aged brains. Abbreviations: BBB, blood-brain barrier; E2, estrogen; RAS, renin-angiotensin system. Figure was produced using Servier Medical Art (http://www.servier.com).

On the basis of the anti-inflammatory effects of IGF-1, it has been suggested that development of resistance to IGF-1 may contribute to neuroinflammation and progression of major brain diseases. Furthermore, neuroinflammation may contribute to disease progression by decreasing levels of neuroprotective molecules such as IGF-1. Therefore, aging-related decrease in IGF-1 levels may contribute to the aging-related pro-inflammatory state and vice versa. However, the molecular mechanisms involved in the above mentioned controversial effects of IGF-1 have not been clarified. Investigation of the specific role of IGF-1 in neurons, astrocytes and microglia in different experimental contexts may shed light on the controversy (Figure 1).

## IGF-1 and Neurons

Our recent studies and others (Zhou et al., 1999; Rodriguez-Perez et al., 2016) have shown the presence of IGF-1 and IGF-1R in neurons, astrocytes and microglia. IGF-1 is synthesized by both neurons and glial cells, although its role is different in each cell type. IGF-1 protects neurons from neurotoxins in the presence of glia, which may be related to direct IGF-1 regulation of the glial inflammatory response (Nadjar et al., 2009). However, IGF-1 may also modulate neuroinflammation indirectly through effects on neurons, which modulate the neuroinflammatory response. IGF-1 directly protects neurons in pure neuronal cultures (Offen et al., 2001; Rodriguez-Perez et al., 2016). The mechanisms responsible for the direct neuronal protection of IGF-1 have not been clarified. However, effects on mitochondrial function (Puche et al., 2008), inhibition of OS, Sirtuin-1 activation (Vinciguerra et al., 2009; Tran et al., 2014) and other possible mechanisms have been suggested (Fernandez and Torres-Alemán, 2012; Torres Aleman, 2012; Werner and Leroith, 2014).

## IGF-1 and Astrocytes

IGF-1 is also synthesized by astrocytes, and several studies indicate that IGF-1 plays a major role in modulating the astrocytic activity. IGF-1 regulates astrocytic glucose control and CNS glucose metabolism (Hernandez-Garzón et al., 2016). Furthermore, astrocytes contribute to regulation of IGF-1R expression in neurons (Costantini et al., 2010). Astrocytic IGF-1 protects neurons against oxidative damage (Genis et al., 2014) and traumatic brain injury (Madathil et al., 2013). IGF-1 administration or IGF-1 gene therapy inhibited expression of toll-like receptor 4, and reduced the astrocytic response to inflammatory stimuli (Bellini et al., 2011) and the expression of inflammatory mediators such as TNF-α, IL-1β and iNOS (Park et al., 2011). However, it was also found that IGF1R-deficient mice showed less amyloid-β oligomer-induced activation of astrocytes and less microgliosis (Cohen et al., 2009; George et al., 2017). Furthermore, astrocytes modulate the inflammatory response both directly and indirectly by releasing mediators that control the microglial response (Dominguez-Meijide et al., 2017).

## IGF-1 and Microglia

Microglia act as resident macrophages in the CNS and major mediators of neuroinflammatory responses (Prinz and Priller, 2014). Classically, microglia present two functional states (i.e., resting and activated microglia). In a healthy state, neurons release immunosuppressive signals, which induce the classical inactivated state in the surrounding microglial cells (Harrison et al., 1998). However, it is now considered that the classical microglial activation comprises a group of “activated” states, and that microglia, in response to their environment, can adopt multiple phenotypes and functions to control CNS homeostasis (Ransohoff, 2016; Labandeira-Garcia et al., 2017; Nissen, 2017). Appropriate progression of the microglial response from the so-called proinflammatory/M1 to the so-called immunoregulatory/M2 phenotype is required for an efficient repair of brain injuries. A dysregulation of this process, leading to continued release reactive nitrogen species (RNS), reactive oxygen species (ROS) and inflammatory cytokines, results in progression neuronal death and brain diseases (Kettenmann et al., 2011; Labandeira-Garcia et al., 2017).

Microglial cells are a major source of IGF-1, which is upregulated during the inflammatory process. IGF-1R are predominantly expressed in neurons and astrocytes, which appear to be targeted by IGF-1 in lesioned regions (Butovsky et al., 2006; Suh et al., 2013) to promote neuronal survival (Arroba et al., 2011; Ueno et al., 2013; Figure 1). The immunoregulatory/M2 phenotype is typically promoted by cytokines such as IL-4 or IL-13. However, a number of data suggest that IGF-1 may also modulate the microglial phenotype: an increase in IGF-1 levels promotes the M2 phenotype (Lee et al., 2013), and treatment with IL-4 increases IGF-1 release by microglial cells (Ferger et al., 2010). IGF-1 also inhibits microglial ROS and markers of M1 phenotype such as TNF-α (Grinberg et al., 2013). Consistent with this, microglia showed a marked upregulation of IGF-1 levels and down-regulation of IL-6 in a transgenic mouse model of amyotrophic lateral sclerosis, which was related to modulation of a beneficial inflammatory response to neuronal damage (Chiu et al., 2008). Similarly, in rat models of ischemic lesion, IGF-1 mRNA was overexpressed in astrocytes and microglia surrounding surviving neurons (Beilharz et al., 1998), and elimination of proliferating microglia enhanced infarct volume and reduced levels of IGF-1 induced by the ischemic lesion (Lalancette-Hébert et al., 2007). Furthermore, microglia may be responsible for the neuroprotective effects of peripheral IGF-1 transported into the CNS, since treatment of microglia with IGF-1 was mitogenic (O’Donnell et al., 2002), and proliferation of microglia is usually considered a major regulator of the neuroinflammatory response, and a source of neuroprotective molecules such as IGF-1 (Lalancette-Hébert et al., 2007). Apparently controversial results were also observed: activation of human microglia with LPS reduced IGF-1 levels, which suggested that chronic neuroinflammation and increased inflammatory cytokines may lead to neurodegeneration by inhibiting the release of microglia-derived neuronal trophic factors, such as IGF-1 (Suh et al., 2013). However, a sudden increase in IGF levels may induce feedback signals decreasing responsiveness of the IGF-1R and IGF signaling, which may be different to the aging and neurodegenerative context (Suh et al., 2008).

Interestingly, aging (Lee et al., 2013) and mitochondrial dysfunctions (Ferger et al., 2010) inhibited IL-4-induced M2 phenotype and IL-4-induced increase in IGF-1 levels. This is consistent with recent studies suggesting that mitochondrial metabolism may modulate microglial polarization (Orihuela et al., 2016).

## Interaction between IGF-1 and Estrogen in Neuroinflammation

Sex hormones induce trophic effects on neurons and glia, enhance neuron survival and modulate several CNS functions (Rettberg et al., 2014). As in the case of IGF-1, estrogens are transported to the brain through the BBB and, in addition, the CNS can synthesize some endogenous estrogens (Azcoitia et al., 2011). Several studies have shown the presence of estrogen receptors (ERs) in neurons, astrocytes and microglia (Rettberg et al., 2014; Rodriguez-Perez et al., 2015). The mechanism responsible for estrogen-induced neuroprotection is still unclear. Direct anti-apoptotic (Brendel et al., 2013) and trophic (Campos et al., 2012) effects on neurons have been observed. However, a number of recent studies have revealed that regulation of the neuroinflammatory response constitutes a major mechanism involved in estrogen neuroprotective effects (Morale et al., 2006; Vegeto et al., 2008).

Several studies have shown that neurons and glial cells co-express ERs and IGF-1R (Cardona-Gómez et al., 2000; Quesada et al., 2007), and that estrogen and IGF-1 interact in the CNS for regulation of developmental and synaptic plasticity events and adult neurogenesis (Garcia-Segura et al., 2010). Furthermore, IGF-1 may mediate neuroprotection induced by estrogen. In aged ovariectomized rats, JB-1 (IGF-1R inhibitor) inhibited the beneficial effects of estrogen administration, suggesting that the IGF-1R signaling may mediate the effects of estrogen (Witty et al., 2013). Similarly, in a rat model of stroke, JB-1 blocked the estrogen neuroprotective effects (Selvamani and Sohrabji, 2010; Sohrabji, 2015). Several possible molecular mechanisms have been involved in the IGF-1/estrogen interaction, such as cross-regulation of ER and IGF-1R expression, IGF-1 regulation of ER-induced transcription, or regulation of IGF-1R signaling by estrogen (Garcia-Segura et al., 2010). Estrogen and IGF-1 may also stimulate common signaling pathways such as MAP kinases, PI3kinase/AKT ERK1 and ERK2 (Singh et al., 1999; Sohrabji, 2015).

Administration of either IGF-1 or estrogen can modify the inflammatory response and microglial and astrocytic phenotypes. However, they may also induce synergistic effects on neuroinflammation by still unclear mechanisms. Brain lesions stimulate production of IGF-1 and estrogen by reactive astrocytes, and increase ER and IGF-1R expression in activated glial cells (Garcia-Estrada et al., 1992; Blurton-Jones and Tuszynski, 2001; García-Ovejero et al., 2002; Hwang et al., 2004). Similar to IGF-1, estrogen has complex effects on the inflammatory response (Bellini et al., 2011; Rodriguez-Perez et al., 2015, 2016). Both estrogen and IGF-1 produced by activated glial cells may act directly on the same glial cells and on the surrounding neurons, modulating both the neuroinflammatory response and neuronal survival. Furthermore, as indicated above for IGF-1, effects of estrogen on neuroinflammation may be in part related to regulation of the BBB, since estrogen-deficient conditions such as menopause or reproductive senescence increase permeability of BBB (Bake and Sohrabji, 2004).

Consistent with the important interactions between estrogen and brain IGF-1 system, gender differences have been observed in several responses mediated by IGF-1 such as effects of exercise (Munive et al., 2016). Furthermore, in a series of studies using cell cultures, young rodents and menopausal rats (Rodriguez-Perez et al., 2010, 2011, 2012, 2015, 2016; Labandeira-Garcia et al., 2016), we have revealed important interactions and mutual regulation between estrogen and brain renin-angiotensin system (RAS). Interestingly, we also observed mutual regulation between RAS and IGF-1 (see below).

## Interaction between IGF-1 and RAS in Neuroinflammation

As in the case of IGF-1, the brain RAS has been associated with longevity, neuroinflammation and aging-related neuronal vulnerability to degeneration (Labandeira-Garcia et al., 2011, 2013, 2017; Labandeira-García et al., 2014). Angiotensin II (AII) is classically considered the main effector peptide of the RAS. AII signaling is mediated via AII type 1 and 2 receptors (AT1R and AT2R). AT2R induce effects that counteract those induced by AT1R stimulation (McCarthy et al., 2013). Overstimulation of the local/paracrine/tissue RAS, through AT1R, leads to OS due to NADPH oxidase overactivation, and triggers inflammatory responses. Consistent with this, RAS overactivation reduces longevity and induces aging-related degeneration in several tissues (Benigni et al., 2009, 2013; de Cavanagh et al., 2015).

Mutual regulation between RAS and IGF-1 was observed in peripheral cells, particularly in vascular smooth muscle cells (Ma et al., 2006; Jia et al., 2011) and cardiomyocytes (Leri et al., 1999; Kajstura et al., 2001). In a recent study, we have investigated possible interactions between both systems in the brain and in the neuroinflammatory process in particular (Rodriguez-Perez et al., 2016), and the results may shed light on the role of IGF-1 in the neuroinflammatory response. We observed reciprocal regulation between RAS and IGF-1: IGF-1 administration decreased RAS activity in neurons and glial cells (i.e., IGF-1 decreased AT1R, increased AT2R and reduced angiotensinogen/angiotensin levels). Inversely, AT1R activation increased IGF-1 and IGF-1R levels in microglia, while AT2R stimulation reduced IGF-1 and IGF-1R expression. AII administration promoted the microglial M1 phenotype via AT1R, which was blocked by activation of AT2R. Consistent with the above-mentioned interactions, the AII-induced enhancement of M1 phenotype markers was inhibited by administration of IGF-1. This suggests that induction of microglial IGF-1 production by AII, and possibly by other OS and pro-inflammatory inducers, plays a major role in the repression the M1 microglial neurotoxic phenotype and the enhancement of the transition to an M2 microglial repair/regenerative phenotype.

According to previous observations in plasma, and other brain regions and tissues (Bartke et al., 2003; Brown-Borg, 2015), we observed a reduction in IGF-1 levels in the substantia nigra of aged rats in comparison with young controls. In young animals and cultures, AT1R stimulation induced an increase in IGF-1 levels (see above). Since AII/AT1R activity is enhanced in the nigra of aged rats (Villar-Cheda et al., 2012, 2014), a counterregulatory increase rather than a decrease in IGF-1 levels may be expected. This suggests an aging-related loss of the IGF-1-mediated counterregulatory mechanism, which may lead to the pro-oxidative and pro-inflammatory state observed aged brains (Villar-Cheda et al., 2012; Lee et al., 2013). Low levels of IGF-1 may contribute to the loss of capacity of microglia to undergo M2 activation in the aged brain.

In addition to IGF-1-induced neuronal protection by modulation of the microglial inflammatory response, IGF-1 mediate a direct effect on neurons (see above), which may lead to indirect (i.e., neuron-mediated) modulation of the glial inflammatory response. This is consistent with our recent studies on the intraneuronal or intracrine RAS, in which we observed that activation of nuclear AT1R induces several intraneuronal protective mechanisms that may counteract the deleterious effects of activation of plasmatic membrane pro-oxidative AT1R (Valenzuela et al., 2016; Villar-Cheda et al., 2017). This protective response includes an increase in transcription of IGF-1. Interestingly, this intracrine protective response was impaired in nuclei isolated from aged brains (Villar-Cheda et al., 2017).

## Conclusion

Experimental manipulation of IGF-1 and IGF-1R led to some controversial findings, possibly because a fine tuning of IGF-1 levels is necessary for each specific situation. This may be critical for regulating the neuroinflammatory response, as well as other IGF-1 functions and brain health. Furthermore, IGF-1 may be involved in inflammation in a context-dependent mode. Views of IGF-1 as beneficial or detrimental appear over-simplistic. Future studies taking into account different experimental contexts and progression in the knowledge of the microglial responses and phenotypes will help to solve current controversies.

## Author Contributions

All authors have contributed to this work and approved its final version for submission. JLL-G developed the idea for this review and wrote the manuscript. AIR-P prepared the figure and was involved in literature review and revision of the manuscript. MAC-B, CML and BV-C were involved in literature review and preparation of the manuscript.

## Conflict of Interest Statement

The authors declare that the research was conducted in the absence of any commercial or financial relationships that could be construed as a potential conflict of interest.
